# Parathyroid Hormone Disturbances in Postmenopausal Women with Distal Forearm Fracture

**DOI:** 10.1007/s00268-021-06331-w

**Published:** 2021-10-13

**Authors:** Axel Wihlborg, Karin Bergström, Paul Gerdhem, Ingrid Bergström

**Affiliations:** 1 Department of Clinical Science, Intervention and Technology, Karolinska Institutet, Solna, Sweden; 2 Department of Orthopedics, K54. Karolinska University Hospital, Huddinge, SE-141 86 Stockholm, Sweden; 3 Karolinska Institutet. Department of Endocrinology, Metabolism and Diabetes, Karolinska University Hospital, Huddinge, 141 86 Stockholm, Sweden; 4 Department of Endocrinology, Metabolism and Diabetes, Karolinska University Hospital, Huddinge, 141 86 Stockholm, Sweden

## Abstract

**Background:**

Primary hyperparathyroidism (PHPT) is a common endocrine disorder with a wide range of adverse effects, such as osteoporosis. Many women are not diagnosed due to asymptomatic disease or vague symptoms but are still at risk of severe adverse effects. Early identification of patients with PHPT is therefore of importance. The aim of this study was to determine PHPT prevalence among postmenopausal women with a distal forearm fracture.

**Methods:**

Recruitment was conducted in conjunction with the occurrence of a distal forearm fracture at Karolinska University Hospital. In total, 161 postmenopausal women were included in a cross-sectional study with repeated evaluations. Analyzes of serum calcium, ionized calcium, phosphate, parathyroid hormone (PTH), and vitamin D were performed. Diagnosis of PHPT was based on clinical evaluations and biochemical definitions of serum calcium and PTH in coherence with previous population prevalence reports.

**Results:**

Mean age was 64.7 (9.5) years, serum calcium 2.33 (0.10) mmol/L, ionized calcium 1.25 (0.05) mmol/L and PTH 54 (26) ng/L. PTH was elevated in 32 (20%) women. In total, 11 (6.8%) women were diagnosed with PHPT; 6 with classical PHPT and 5 with mild PHPT. The prevalence of PHPT was significantly increased compared to the population prevalence of 3.4% (*p* = 0.022).

**Conclusion:**

Screening postmenopausal women in conjunction with low-energy distal forearm fracture revealed a large number of women with parathyroid disturbance. Evaluation of parathyroid hormone and calcium status in this group of patients seems beneficial.

## Introduction

Primary hyperparathyroidism (PHPT), a common endocrine disorder among postmenopausal women, can result in a wide range of adverse effects, including severe disturbance of calcium metabolism and osteoporosis, a condition that increases fracture risk [1]. Around 20% of PHPT patients suffer from osteoporosis, renal stones, and/or psychological and neuromuscular symptoms [1]. Parathyroidectomy increases bone mineral density and reduces fracture risk even in mild cases of PHPT [2–6], reduces the incidence of renal stones, and improves psychological as well as neuromuscular symptoms [2, 5, 7]. Parathyroidectomy also reduces fracture risk in most cases of asymptomatic PHPT [3, 4, 8, 9]. According to guidelines, asymptomatic PHPT should be treated with parathyroidectomy if other risk factors for fracture such as osteoporosis are present [10]. When these surgical indications are not met, long-term surveillance is recommended [10, 11].

A variant of PHPT, normocalcemic PHPT, is characterized by elevated PTH in combination with calcium within the upper reference range. Normocalcemic PHPT may be associated with osteoporosis and renal stones despite normal calcium values and may be a precursor of classical PHPT [1, 10, 12, 13]. Elevated PTH in PHPT increases bone resorption, and the catabolic effect of PTH has been associated with both cortical and trabecular deterioration [14–16]. Among fracture patients, however, PHPT prevalence is not well described. While it is reasonable to assume that PHPT is more frequent in patients with osteoporosis-related fractures, studies have reported conflicting results [17–20].

Previously, we found that elderly women with a distal forearm fracture had an increased prevalence of PHPT [21], confirming the results from Mallmin et al. [22]. For this reason, Mallmin et al. proposed screening for PHPT in women with a distal forearm fracture who are older than 60. However, to the best of our knowledge, no additional studies regarding PHPT among postmenopausal women with distal forearm fracture have been conducted. The absence of general guidelines regarding screening of this population implies that further studies are needed.

Adequate diagnosis of PHPT requires repeated biochemical and clinical evaluations. This cross-sectional study aimed to determine the prevalence of classical and mild PHPT among postmenopausal women with a distal forearm fracture using repeated clinical and biochemical evaluations.

## Materials and methods

The study was initiated in 2010 at Karolinska University Hospital in Stockholm, Sweden. Postmenopausal women presenting with a distal forearm fracture at the orthopedic emergency department between April 2010 and January 2015 giving informed consent were included in the study. All fractures were radiologically confirmed and were caused by low-energy trauma, defined as falling from a standing height or less.

In total, 161 women were included at the emergency ward and screened with blood sampling. In addition, all women visited the research facility for supplementary blood sampling. The women were subsequently invited to the department of endocrinology for additional evaluation with a complete biochemical analysis and physical examination. Additional evaluations were performed in case of suspected PHPT or other calcium metabolism disturbances. All suspected cases of PHPT were confirmed by a consultant endocrinologist without affiliation to the study. Elevated PTH or hypercalcemia due to secondary causes; Vitamin D deficiency, malabsorption, concomitant medication or disease including familial hypocalciuric hypercalcemia (FHH) or a serum creatinine level above 90 µmol/L were disregarded in terms of PHPT. None of the patients with PHPT received antiresorptive treatment, calcium, or Vitamin D supplements at screening or during follow-up. Patients with confirmed PHPT were treated in accordance with the regional clinical guidelines in Stockholm (Appendix 1). Women with symptomatic disease, osteoporosis, or kidney stones were referred to the endocrine surgery department for assessment regarding parathyroidectomy. Follow-up was applied if surgery was not considered appropriate. Fractures and uncovered diseases were treated according to clinical routine.

The biochemical definition of PHPT was designed with a combination of serum calcium and PTH, similar to previous reports of PHPT in Scandinavia [23, 24]. Serum calcium >2.60 mmol/L in combination with PTH >35 ng/L; serum calcium 2.50–2.60 mmol/L in combination with PTH >45 ng/L or serum calcium <2.50 mmol/L (but in the upper part of the reference range 2.15–2.50 mmol/L) in combination with PTH > 65 ng/L were, in the absence of secondary causes, consequently considered as primary hyperparathyroidism. Thus, meeting the biochemical definitions would, like previous population prevalence reports entail both classical PHPT as well as mild PHPT [23, 24]. In addition, ionized calcium was assessed as a complement to the calcium status evaluation. Reevaluations were performed, similar to previous population prevalence reports [23, 24], as patients with PHPT may display fluctuating serum calcium values with occasional values within the normal reference range [25] and in order to exclude secondary causes.

The women diagnosed with PHPT were subdivided into groups of classical PHPT and mild PHPT. Classical PHPT was defined as simultaneously elevated serum calcium (>2.50 mmol/L) and PTH (>65 ng/L) in the 1st, 2nd, or 3rd sample. Mild PHPT was defined as elevated serum calcium with PTH within the upper part of the reference interval in either 1st, 2nd, or 3rd sample, combined with elevated PTH and serum calcium in the upper reference interval at some point during follow-up. Mild PHPT also comprised normocalcemic PHPT with elevated PTH in either 1st, 2nd, or 3rd sample, combined with persistent serum calcium and ionized calcium within the upper part of the reference interval.

All parts of the study were approved by the Regional Ethical Board in Stockholm (2009/913–31).

### Bone mineral measurements

In each subject, the areal bone mineral density of the left femoral neck, the left total hip, the lumbar spine, and the contralateral forearm (33% of the radial bone) were measured using DXA (GE Lunar iDXA, GE Medical Systems, Chalfont St. Giles, UK). Calibration was performed daily, combined with weekly calibrations with spine phantom provided by the manufacturer. CV% for the spine phantom was 1.5%. Data from the left hip and first and second lumbar vertebra was used [26].

### Biochemical and statistical analyses

Biochemical analysis was performed as part of the clinical routine. The specified reference range in text refers to the current reference range at the Hospital. Additional serum was stored at − 80 °C. Serum calcium (reference range: 2.15–2.50 mmol/L) was determined by oCPC procedure up to 2012 (intra- and interassay CV% 0.9 and 1.1–4.8) and subsequently by NM-BAPTA method (intra- and interassay CV% 1.6 and 1.8–8.6), Roche diagnostics Ldt. Serum calcium was corrected for albumin levels (reference range: 34–45 g/L) by Calcium + 0.01 x (39-albumin). Serum calcium in text refers to serum calcium corrected for serum albumin. Ionized calcium (reference range: 1.15–1.33 mmol/L) was measured on an ABL 800 FLEX, Radiometer, A/S Copenhagen, Denmark. Parathyroid hormone, including PTH from frozen serum, (reference range: 10–65 ng/L) was measured by the use of Electrochemiluminescence immunoassay (ECLIA) as intact PTH on Modular Analytics E170 until October 14, 2015 (intra- and interassay CV% 1.1–2.0 and 2.8–3.4). From October 15, 2015 PTH was analyzed as PTH 1–84 on Cobas e602 analyzer, Roche diagnostics Ldt (intra- and interassay CV% 0.7–3.5 and 3.0–6.2). Regression analysis has shown a good correlation between the methods (*r* = 0.99). In cases of missing PTH value at screening, PTH and serum calcium were assessed separately from the frozen serum (*n* = 51). Vitamin D was measured as 25-hydroxy vitamin D3 (25OHD) (nmol/L) from the frozen serum with API 4000 LC–MS/MS system, Sciex (CV% 4.0–6.0).

Descriptive data is presented as mean (SD). Binomial two-tailed exact test was used to determine the difference in PHPT prevalence from population prevalence. A *p*-value <0.05 was regarded as significant. With a population prevalence of 0.02, a study population of 141 women was needed, assuming a prevalence of 0.06 in the study population as seen in our earlier study results [17] (α = 0.05, power 80%). Statistical analyzes were performed with IBM SPSS Statistics version 20.

## Results

**Fig. 1:**
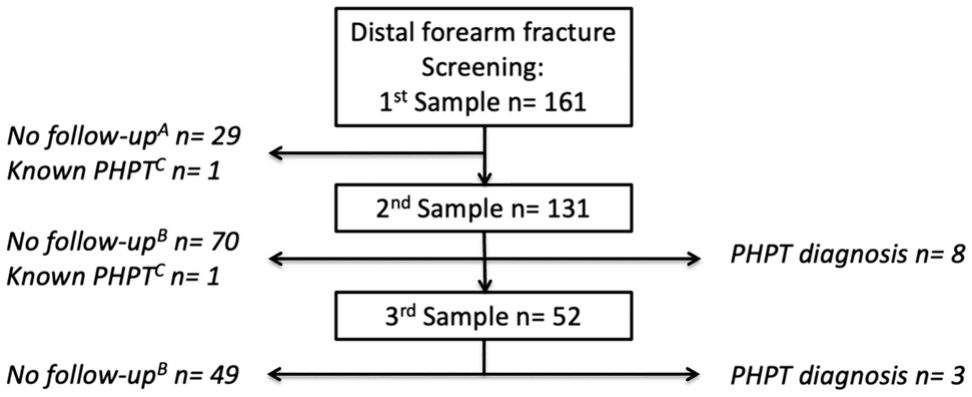
Flowchart of the screening and follow-up procedure. The 2nd blood sample included a full clinical evaluation. A total of 11 women were diagnosed with PHPT after the 2nd and 3rd samples. PHPT = Primary hyperparathyroidism. **a** Declined or no follow-up available. **b** No further follow-up indicated. **c** Already diagnosed with PHPT at the time of fracture

**Fig. 2:**
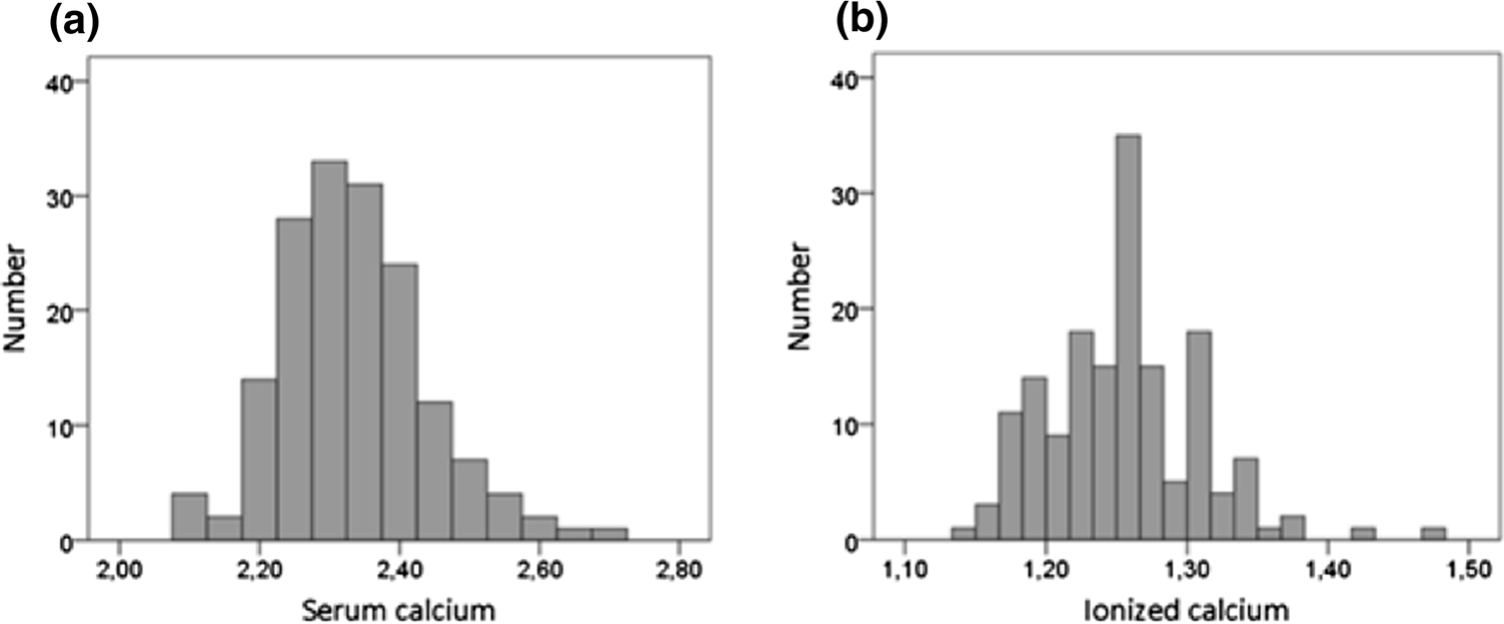
Distribution at screening of the 161 women with a distal forearm fracture. **a** Serum calcium corrected for serum albumin (mmol/L). **b** Ionized calcium (mmol/L)

In total, 161 women with a low-energy distal forearm fracture were included at a mean age of 64.7 (9.5) years. The mean time from fracture to the 1st blood sample was 4 (17) days and 137 women (84%) were analyzed on the same day as the fracture. Mean time from fracture to the 2nd and 3rd blood samples was 4.0 (3.0) months and 7.1 (3.1) months, respectively (Fig. 1). Mean serum calcium was 2.33 (0.10) mmol/L and ionized calcium 1.25 (0.05) mmol/L at screening (Fig. 2). Mean PTH was 54 (26) ng/L and 25OHD 63 (22) nmol/L at screening. One woman was already diagnosed with PHPT when the fracture occurred, and one woman had a relapse of previously surgically treated PHPT. These two women were excluded from the analysis.

Out of the 161 women, 11 (6.8%) were diagnosed with PHPT during the follow-up. The biochemical profiles at screening and during follow-up of the 11 women diagnosed with PHPT are shown in Table 1. Classical PHPT, with both serum calcium and PTH values greater than the reference range (>2.50 mmol/L and > 65 ng/L, respectively) in the 1st, 2nd, or 3rd sample was found in 6 women (Table 1, patients no 1–6). Mild PHPT, with PTH values greater than the reference range (>65 ng/L) and serum calcium values in the upper part of the reference interval in the 1st, 2nd, or 3rd sample was found in 5 women (Table 1, patients no 7–11). Of these, one woman presented with normocalcemic PHPT, i.e., elevated PTH combined with persistent calcium levels within the upper part of the reference interval (Table 1, patient no 11). Among the women with mild PHPT, hypercalcemia with PTH within the upper part of the reference range was present in 3 women at some time during follow-up (Table 1, patients no 7–9). Ionized calcium was elevated (>1.33 mmol/L) in all the women with classical PHPT and in 2 of the 5 women with mild PHPT, in the 1st, 2nd, or 3rd sample (Table 1, patients no 1–6; 9–10).

**Table 1:** Biochemical profile of the 11 women diagnosed with PHPT. Serum calcium corrected for serum albumin. 25-hydroxy vitamin D (25OHD) was assessed separately from plasma stored at screening. PTH and serum calcium at screening were assessed from the supplementary samples in patients no 4–6 and 9. Mean time to 2nd sample: 4.4 (3.2) months and 3rd sample: 5.9 (3.7) months. Reference range: serum calcium (2.15–2.50 mmol/L), ionized calcium (1.15–1.33 mmol/L), phosphate (0.80–1.50 mmol/L), PTH (10–65 ng/L). NA = not available. Ca = calcium. PTH = parathyroid hormone

	Patient	Age	1st sample (Screening at fracture)					2nd sample			3rd sample		
			Serum Ca	Ionized Ca	PTH	Phosphate	25OHD	Serum Ca	Ionized Ca	PTH	Serum Ca	Ionized Ca	PTH
	*No*	*Years*	*mmol/L*	*mmol/L*	*ng/L*	nmol/L	*mmol/L*	*mmol/L*	*mmol/L*	*ng/L*	*mmol/L*	*mmol/L*	*ng/L*
*Classical PHPT*	1	60	2.54	1.43	76	0.75	71	2.61	1.46	NA	2.60^A^	1.43	71
	2	71	2.47	1.28	204	0.95	59	2.66	1.44	191	2.84	1.52	NA
	3	57	2.37	1.34	104	0.70	57	2.50	1.35	58	2.52	1.37	82
	4	77	2.55	1.35	77	0.87	47	2.58	1.39	81	2.51	1.34	89
	5	61	2.67	1.36	113	0.73	56	2.51	1.40	91	2.54	1.42	104
	6	69	2.64	1.37	78	0.92	56	2.44	1.40	91	2.41	1.40	88
*Mild PHPT*	7	57	2.49	1.29	70	0.82	67	2.37	1.28	67	2.68	1.29	59
	8	59	2.51	1.31	58	0.90	51	2.47	1.30	70	2.58^A^	1.30	60
	9	50	2.53	1.37	53	0.94	85	2.46	1.42	72	2.23	1.34	62
	10	63	2.48	1.34	58	0.92	95	2.36	1.31	67	2.41^A^	1.29	78
	11	62	2.46	1.30	48	0.89	52	2.41	1.31	72	2.49	1.28	70

^A^Serum calcium not corrected for serum albumin

Clinical profiles and T-scores of the 11 women diagnosed with PHPT are presented in Table 2. Symptomatic disease, defined as the presence of fatigue, muscle weakness, joint pain, kidney stones, or depression was found in 3 women with classical PHPT and in 2 women with mild PHPT. Osteoporosis (T-score ≤ − 2.5) and a history of kidney stones were found in one woman (patient no 6). Forearm T-score ≤ 2.5 (33% of the radial bone) was found in 3 women (patients no 1, 6, 11). None of the women had previously been evaluated by an endocrinologist. Of the 6 women with classical PHPT, 3 were recommended surgery. Of the 5 women with mild PHPT, 2 were recommended surgery. Parathyroid gland adenoma was found in all the surgically treated women.

**Table 2:** DXA results and clinical profiles of the 11 women with diagnosed PHPT. Clinical feature refers to the presence of symptoms such as fatigue, muscle weakness, joint pain, or depression. NA = not available

	Patient *No*	Total hip *T-score*	Femoral neck	L1-L2	Osteoporosis	33% of Radius	Clinical feature	Kidney stone	Clinical outcome
			*T-score*	*T-score*	*T-score* < *-2.5*	*T-Score*			
*Classical PHPT*	1	− 1.1	− 1.2	− 2.4	No	− 2.5	Asymptomatic	No	Follow-up
	2	− 1.2	− 1.7	− 0.8	No	− 1.4	Symptomatic	No	Surgery
	3	− 2.2	− 2.2	− 2.3	No	− 0.2	Asymptomatic	No	Follow-up
	4	− 2.0	− 2.4	− 2.1	No	− 1.3	Asymptomatic	No	Follow-up
	5	− 1.1	− 1.7	− 0.8	No	NA	Symptomatic	No	Surgery
	6	− 1.6	− 1.8	− 3.2	Yes	− 5.0	Symptomatic	Yes	Declined surgery (Follow-up)
*Mild PHPT*	7	− 1.4	− 2.4	− 1.2	No	− 0.6	Asymptomatic	No	Follow-up
	8	− 1.9	− 1.7	− 1.6	No	− 1.4	Symptomatic	No	Declined surgery (Follow-up)
	9	− 0.9	− 1.7	0.1	No	− 0.5	Asymptomatic	No	Follow-up
	10	− 0.9	− 1.2	− 1.9	No	− 1.9	Asymptomatic	No	Follow-up
	11	− 2.1	− 2.1	− 2.1	No	− 3.2	Symptomatic	No	Surgery

The PHPT prevalence of 6.8% (11 women) was significantly increased compared to the population prevalence of 3.4% (*p* = 0.022). Population prevalence has previously been reported to range between 1.36 and 3.4% [23, 24, 27].

**Fig. 3:**
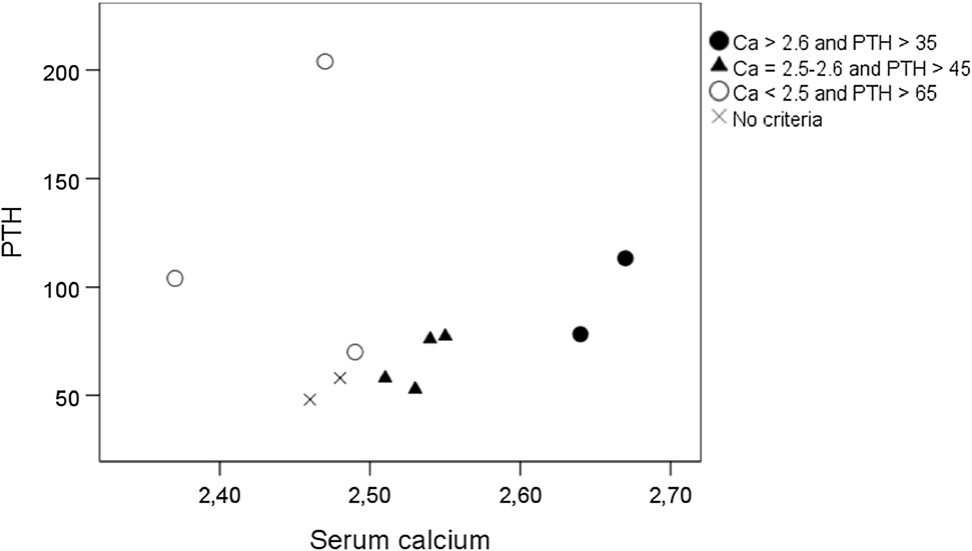
Serum calcium corrected for serum albumin (mmol/L) and PTH (ng/L) at screening of the 11 women diagnosed with PHPT during follow-up. Arranged according to the biochemical definition of PHPT at screening. No criteria = did not meet any of the biochemical definitions of serum calcium and PTH at screening

The relationship between PTH and serum calcium at screening in the 11 women with PHPT is depicted in Fig. 3. Of the 11 women diagnosed with PHPT, 9 women met any of the biochemical definitions at screening (Table 1, patients no 1–9).

PTH was elevated above the reference range (>65 ng/L) in 32 of the 161 women (20%) at screening (Table 3). Of these, 9 women had elevated PTH due to PHPT, leaving 23 women with elevated PTH for reasons other than PHPT. The reasons for elevated PTH in these 23 women were; Vitamin D insufficiency <50 nmol/L, vitamin D deficiency < 25 nmol/L, serum creatinine above 90 μmol/L, and severe liver cirrhosis. Two of the women declined further follow-up.

**Table 3:** List of reasons for PTH elevated above the reference range (> 65 ng/L) at screening

	Number of women
Elevated PTH at screening (>65 ng/L)	32
PHPT	9
Vitamin D insufficiency <50 nmol/L	15
Vitamin D deficiency <25 nmol/L	2
Serum creatinine >90 μmol/L	3
Severe liver cirrhosis	1
Declined further follow-up	2

## Discussion

Of the 161 postmenopausal women with a distal forearm fracture, we identified 11 women (6.8%) with primary hyperparathyroidism. Six of these women exhibited classical PHPT and five mild PHPT. All the 11 women met the biochemical definition and the diagnostic criteria for PHPT, as defined by previous population prevalence reports [23, 24]. The PHPT prevalence was significantly greater than previously reported population prevalence in women of similar age in Nordic countries, ranging between 1.34 and 3.4% [23, 24, 27].

These results suggest that postmenopausal women with low-energy distal forearm fractures are more likely to have parathyroid hormone disturbances, a finding that agrees with some previous studies [21, 22]. Similarly, previous studies have reported an increased PHPT prevalence in elderly patients following a hip fracture or fracture in general [17–20]. On the other hand, Melton et al. were unable to demonstrate an increased occurrence of PHPT among women with distal forearm fractures. However, their retrospective study design was based solely on the review of medical records, a methodology that makes the diagnosis of asymptomatic or mild PHPT less reliable than our methodology [28]. Comparisons between studies are precarious because of differences in study design and biochemical definitions of PHPT. Furthermore, not all studies identify PHPT by clinical examinations and repeated measurements, which were performed in our study. Our findings implicate that further studies with a similar methodology could highlight possible correlations with other low-energy fractures. Increased prevalence of PHPT has been suggested among elderly patients with a hip fracture [17, 19] and further studies regarding PHPT prevalence in this group of patients would be of interest.

All of the women diagnosed with PHPT suffered from low bone mass (*T*-score ≤–1.0) and one of the women had osteoporosis based on *T*-score ≤−2.5. However, clinical osteoporosis was present in all women diagnosed with PHPT based on the presence of a low-energy fracture and low bone mass. Other clinical symptoms that might be correlated with PHPT were found in 5 women. Although some biochemically and symptomatically mild cases of the disease were revealed in this study, identifying these women is advantageous as progression may occur and long-term surveillance is advised even in mild cases [1, 10–13]. This study revealed normocalcemic PHPT in 1 out of 161 women (0.62%) with a distal forearm fracture, which is similar to the previously shown prevalence of 0.74% among adults without a previous fracture or nephrolithiasis [29].

We found a relatively high proportion of women (20%) with elevated PTH (>65 ng/L). The deteriorative effects of PTH on both cortical and trabecular compartments of the radial bone in women with PHPT as well as in women with elevated PTH for reasons other than PHPT, have previously been demonstrated [15, 16, 30, 31]. However, the present study does not allow for a direct assessment of this association.

The fracture risk seems to be increased in postmenopausal women following a low-energy distal forearm fracture and a subsequent clinical evaluation in terms of osteoporosis is generally indicated [32]. Our findings suggest that screening of calcium and PTH levels in this group of patients could complement the evaluation in terms of parathyroid disturbances. Although screening in this study was performed at the emergency ward, it seems preferable to perform this in a calmer stage by primary care physicians or within the context of a fracture risk assessment.

This study has several strengths. It is based on a large cohort of postmenopausal women with a low-energy distal forearm fracture. All women were recruited consecutively and the cohort size was predetermined in accordance with the power analysis. The participants were clinically evaluated with longitudinal follow-up, and the diagnoses were confirmed by external endocrinologists as well as endocrine surgeons. The diagnosis was determined by clinical visits and biochemical reevaluations, and we used similar diagnostic criteria as recent population prevalence reports in order to intercept both classical and mild PHPT.

The study design has a number of limitations. Few studies have examined PHTP prevalence in similar settings and these studies vary in design, screening methods, and biochemical definitions. Some population prevalence reports are based on screening serum calcium solely, which impedes precise comparisons. A control group would have strengthened the study. However, the required sample size would have made the study difficult to implement. In total, 168 women participated at screening at the emergency ward, of which 7 women declined further visits to the research center. The inclusion rate was slower than expected due to interruptions in study recruitment caused by personnel and workplace changes at the emergency ward. Still, few women declined participation. Therefore, we believe the risk of recruitment bias is low. PTH was missing at screening in a few women, but stored samples were available. Not all cases were surgically treated and diagnosis confirmation by pathological examination was consequently not available for all. A small subset of women did not participate in the clinical evaluation, which might be a source of underestimation in regards to the PHPT prevalence in the study population.

## Conclusions

In summary, a large number of postmenopausal women with a recent low-energy distal forearm fracture were diagnosed with PHPT. In addition, a large proportion of the study population exhibited elevated PTH for reasons other than PHPT. Whether these findings are applicable in different settings or within different ethnical groups cannot be determined from the present study, and further research is needed in order to affirm associations.

Distal forearm fracture is one of the most common fractures in postmenopausal women and the high presence of parathyroid disturbance suggests that evaluation of parathyroid hormone and calcium status could be of benefit in this group of patients.

## Regional clinical guidelines in Stockholm, Sweden

Excerpt from the Regional clinical guidelines regarding referral of patients with primary hyperparathyroidism (pHPT) to the endocrine surgery department. Translated 2021-09-07 from www.viss.nu/kunskapsstod/vardprogram/hypercalcemi, originally published in 1999 and updated continuously.

Responsible for the clinical guidelines are the Stockholm-Gotland medical advisory board for endocrinology and diabetology and the Stockholm board pharmaceutical committee for endocrinological and metabolic disorders.

Recommendation for surgery (Parathyroidectomy):

- Osteoporosis (T-score < -2.5 in the distal radius, lumbar spine or hip) or vertebral fracture
- Kidney stone or nephrocalcinosis
- Neurocognitive or neuropsychiatric symptoms that are attributable to pHPT
- Age < 50
- Renal impairment that is attributable to pHPT
- Pancreatitis without other causative factors
- Serum calcium > 2.75 mmol/L in repeated measurements
- Conservative follow-up is expected to be difficult to implement

### Elderly patients

- Higher serum calcium levels and/or more obvious symptoms are required to justify surgery in elderly patients with other serious illnesses which entails an increased risk of surgery.
- Hypercalcemic crisis constitutes an absolute surgical indication. With other indications, the patients’ own preferences weigh heavily. However, there is no alternative treatment to surgery except in exceptional cases where treatment with Cinacalcet can be considered.
- If surgery is not appropriate, treatment with bisphosphonates or denosumab may be considered for skeletal protection.

### Younger patients

- In younger patients (< 50 years), surgery should be considered in all patients with suspected pHPT due to the risk of future morbidity, including a likely increased risk of cardiovascular disease.
